# Nutritional variation in baobab (*Adansonia digitata* L.) fruit pulp and seeds based on Africa geographical regions

**DOI:** 10.1002/fsn3.502

**Published:** 2017-08-27

**Authors:** Kinuthia U. Muthai, Mbuthia S. Karori, Alice Muchugi, Abwao S. Indieka, Catherine Dembele, Simon Mng'omba, Ramni Jamnadass

**Affiliations:** ^1^ Biochemistry and Molecular Biology Department Egerton University Egerton Kenya; ^2^ World Agroforestry Centre (ICRAF) Nairobi Kenya; ^3^ World Agroforestry Centre (ICRAF) Bamako Mali; ^4^ World Agroforestry Centre (ICRAF) Lilongwe Malawi

**Keywords:** baobab, minerals, proximate composition, pulp, seeds, variation

## Abstract

Baobab (*Adansonia digitata* L.) is an indigenous fruit tree associated with the Savannah drylands of sub‐Saharan Africa. Local communities mainly utilize the leaves, pulp, and seeds of baobab as a source of food and for income generation. The present study was conducted to determine the nutritive attributes of baobab fruit pulp and seeds across provenances in east, west, and southern Africa and to determine whether the nutrient content varied with the provenance of origin. Pulp and seed proximate composition and mineral element concentration were determined using the AOAC 1984 methods and inductively coupled plasma atomic emission spectroscopy (ICP‐AES), respectively. The results showed that there exist significant variation (*p* < .05) in pulp moisture, protein, fiber, ash, and elemental content among provenances. The highest mean pulp crude fiber (8.68 g 100 g^−1^ dw) was recorded in Kenya. At country level, Malawi had the highest mean pulp potassium (22.2 mg g^−1^), calcium (4,300 mg kg^−1^), magnesium (2,300 mg kg^−1^), sodium (1,000 mg kg^−1^), and phosphorus (1,100 mg kg^−1^) levels. Kenya had the highest mean pulp iron (57.4 μg g^−1^) and manganese (27.2 μg g^−1^) content, while Mali had the lowest iron (13.1 μg g^−1^) and manganese (8.6 μg g^−1^). At country level, the mean seed calcium content was highest (3,200 mg kg^−1^) in Malawi and lowest (2,000 mg kg^−1^) in Kenya. The highest mean iron content of 63.7 μg g^−1^ was recorded in seeds from Kenya, while the lowest (25.8 μg g^−1^) was in Mali. Baobab seed mineral and proximate content varied significantly (*p* < .001) among the selected countries. Overall, baobab fruit pulp and seeds contain significant amounts of nutritionally essential minerals and proximate components but the amounts varied significantly among the selected countries. This variation offers opportunities for selecting provenances to concentrate on during germplasm collection for conservation and domestication of baobab.

## 1 INTRODUCTION

African baobab (*Adansonia digitata* L., Malvaceae) is an important indigenous fruit tree species important for food security, nutrition, and income generation for the rural population in Africa (Chadare, Linnemann, Hounhouigan, Nout, & Van Boekel, 2009; Sidibé & Williams, 2002). Baobab occurs in the drylands of sub‐Saharan Africa, and it is a representative of the wooden “Big Five,” which also include *Tamarindus indica*,* Zizyphus mauritiana*,* Sclerocarya birrea*, and *Mangifera indica* (Jama, Mohamed, Mulatya, & Njui, 2008). The edible parts of baobab (leaves, seeds, and fruit pulp) are consumed mostly by rural communities who also sell them in local markets, whereas the nonfood parts (timber, fodder, and fibers) are mainly used for income generation in sub‐Saharan Africa (Gebauer et al., 2016). Moreover, the species has several roles in traditional medicine and cultural and religious beliefs, which often consider the tree as sacred (Kamatou, Vermaak, & Viljoen, 2011). The naturally dry fruit pulp is rich in vitamin C, calcium, potassium, and dietary fiber (Assogbadjo, Chadare, Kakaï, Fandohan, & Baidu‐Forson, 2012; Gebauer, El‐Siddig, & Ebert, 2002; Simbo et al., 2013; Stadlmayr, Charrondiere, Eisenwagen, Jamnadass, & Kehlenbeck, 2013). The pulp is usually used in the preparation of fruit juice, snacks, sweets, as a fermenting agent in local brews (Gebauer et al., 2014), porridge, and in food recipes (Sidibé & Williams, 2002).

In West Africa, the young tender leaves are commonly consumed fresh as a substitute for commercial vegetables or used to prepare sauces or even as dried powder (Gebauer et al., 2002; Sidibé & Williams, 2002). Baobab seeds are roasted and eaten, or they are used as thickening agents when powdered, or as flavor enhancers when fermented. In Burkina Faso, the fermented baobab seeds are commonly known as *Maari*, are rich in fatty acids and essential amino acids, and form part of the local diet (Parkouda, Ba, Ouattara, Tano‐Debrah, & Diawara, 2015). Baobabs play an important role in providing a balanced nutrition because their edible parts supply vitamins, mineral, proteins, and energy that are not commonly obtained from the cereal‐dominated diets of drylands of Africa.

Several nutritional studies have been conducted on baobab, but there exists a great variation in the reported data depending on the source of fruits and analytical methods used (Chadare et al., 2009; Stadlmayr et al., 2013). Assogbadjo et al. (2012) reported the influence of soil characteristics on the biochemical composition of baobab parts across three agro‐climatic zones of Benin. In Mali, topsoil characteristics and tree provenance were reported to influence the nutritive composition of baobab fruit pulp (Simbo et al., 2013). Although baobabs are commonly available in east and southern Africa, information on their nutritional composition is scarce or fragmented. Moreover, the influence of tree provenance on the nutritive content of baobab seeds and fruit pulp from these regions has not been reported in the literature. The availability of comprehensive nutritional data can significantly promote the consumption of this fruit among many communities. Therefore, the aim of this study was to explore the proximate composition and mineral content of baobab fruit pulp and seeds in East, South, and West African countries and to determine whether the region of origin has an effect on the nutrient content. The information generated from this study will be useful in implementing policies to reduce hunger, improve nutrition, enable value addition of baobab products, and boost economic welfare of rural communities in sub‐Saharan Africa. Moreover, the existence of variation in nutritive attributes across the countries would serve as an opportunity for the selection of superior mother trees from the existing agroforestry systems for domestication, breeding, and conservation.

## 2 MATERIALS AND METHODS

### 2.1 Baobab sampling and processing

Mature, unblemished baobab fruits used in this study were obtained from populations established in 17 provenances (Table 1) across six African countries (Kenya, Tanzania, Zambia, Zimbabwe, Malawi, and Mali). Because genetic differentiation was found to exist between baobab populations from different climatic zones/provenances (Assogbadjo, Kyndt, Sinsin, Gheysen, & Van Damme, 2006), sampling was done randomly without consideration of tree fruit density, fruit size, leaf canopy, or tree height to identify 2–7 trees in each provenance. From each tree, a composite bulk sample (both for pulp and seed) comprised 2–5 fruits from the same tree. In this study, a baobab population was defined as a group of baobab trees randomly and naturally distributed in an agroforestry system. The location (latitude and longitude) of sampled trees from the baobab populations was recorded with the aid of a geographical positioning system (GPS). Baobab fruits were cracked open using a hammer, and fruit pulp detached from the seeds by the use of a mortar and pestle. The pulp was then separated from fibers and seeds by passing them through a 2‐mm sieve. About 20 g of each composite bulk for the pulp was weighed, transferred to labeled zip‐lock bags, then wrapped with aluminum foil before storing them in a refrigerator at 4°C prior to analysis. Seeds from the composite bulk of each tree were soaked in water for almost 1 hr and then washed by hand to remove the remaining pulp and fibers. The seeds were spread on labeled drying trays covered with an adsorbent (paper towels) and left overnight on the laboratory bench to lose moisture acquired during washing. When felt dry, they were packed in cotton bags and stored in a room at 15% relative humidity and 15°C for almost 7 days before milling them into powder using a laboratory mill (Thermo Scientific^™^, USA). The hopper, rotor, blades, and sieve of the mill were cleaned by blowing with air at high velocity and then wiping the parts with 70% ethanol to avoid sample cross contamination.

**Table 1:** The geographical conditions for baobab provenances in East, South, and West Africa

Country	Region	Provenance	Latitude	Longitude	Altitude (m)	Annual temperature (°C)	Annual rainfall (mm)
Kenya	East Africa	Kibwezi	2° 24′ S	37° 58′ E	885	23.0	626
		Taita Taveta	3° 23′ S	38° 33′ E	760	23.3	616
		Malindi	3° 13′ S	40° 07′ E	8	26.3	1,094
Tanzania	East Africa	Moshi rural	4° 30′ S	37°00′ E	730	23.5	916
		Kongwe	6° 12′ S	36° 27′ E	1,114	22.7	579
		Kilolo	7° 28′ S	36° 30′ E	535	19.1	661
		Mwanga	4° 45′ S	37° 36′ E	740	23.1	553
Mali	West Africa	Mopti	14° 29′ N	4° 11′ W	321	28.4	463
		Kayes	14° 26′ N	11° 26′ W	80	29.3	632
		Seguo	13° 25′ N	6° 14′ W	300	28.0	611
		Sikasso	10° 35′ N	7° 26′ W	393	26.9	1,125
Zambia	Southern Africa	Chirundu	16°0′ S	28° 51′ E	458	25.2	656
		Siavonga	16° 26′ S	28° 41′ E	525	25.5	712
		Mambwe	13° 20′ S	31° 49′ E	555	25.1	768
Zimbabwe	Southern Africa	Hwange	19° 25′ S	26° 30′ E	330	24.0	576
		FRC	19° 48′ S	32° 52′ E	400	18.4	831
Malawi	Southern Africa	Mangochi	14° 14′ S	35° 04′ E	470	24.3	841

### 2.2 Biochemical analyses

#### 2.2.1 Proximate analysis

The pulp and seed proximate composition was determined using the methods of AOAC (1984). Moisture content was obtained by difference weighing of 2 g of the sample before and after oven‐drying (model 600; Memmert, Schwabach, Germany) at 105°C for 12 hr. Crude protein was estimated using the macro‐Kjeldahl method, then calculated by multiplying the measured nitrogen by a factor of 6.25. Ash was determined by incinerating the dried sample (2 g) in a muffle furnace (Gallenkamp, England) at 550°C for 6 hr. A sample (10 g) was used to determine crude fat by extracting with n‐hexane (40–60°C) in a Soxhlet apparatus. The crude fiber was determined by digesting the defatted sample (2 g) in 1.25% HCl and 1.25% NaOH. Carbohydrate levels were calculated by subtracting the total sum of moisture, crude protein, crude fat, and ash from 100%.

#### 2.2.2 Mineral element analysis

Approximately 0.5 g of each test sample was ashed in a muffle furnace (Gallenkamp, England) at 460°C for 4½ hr. After cooling, the ashes were then digested by adding a mixture of hydrochloric acid (37%, 0.4 ml), nitric acid (69%, 0.4 ml), and peroxide (30%, 0.6 ml). The digestion was performed by heating the specimen tubes on an electric hotplate to near dryness. After cooling, the digest was treated with 25 ml of 0.05 N HCl and then the specimen tubes were stoppered, shaken thoroughly, and left overnight. For analysis, 2 ml of each digested sample was pipetted into a 25‐ml volumetric flask, then diluted with 5 ml of distilled water before addition of 2.5 ml of 5% strontium chloride. The solution was made up to the 25 ml mark with distilled water. Calcium, potassium, magnesium, sodium, phosphorus, copper, iron, zinc, and manganese were determined using inductively coupled plasma atomic emission spectrometer (ICPE 9000, Shimadzu, Japan). Sample concentrations of each element were determined by comparing their absorbance to a standard linear regression curve from standard solutions.

### 2.3 Statistical analyses

All determinations were conducted in triplicate, and the data were subjected to analysis of variance (ANOVA) using the nested model. In the model, trees were nested in provenances, while provenances were considered as nested factors within countries. Tukey's test was used to identify significant differences between the provenances and countries at α = 0.05 level using GenStat software version 15.1 (VSN International Limited, 2012). Data are presented as mean ± standard error of the mean (*SEM*).

## 3 RESULTS AND DISCUSSION

### 3.1 Proximate composition related to the region of origin

The moisture, crude fiber, crude protein, and ash content of baobab fruit pulp from the five countries are shown in Table 2. Results showed that there were statistically significant differences (*p* < .001) among the countries for the mean pulp moisture, crude protein, crude fiber, and ash content. Baobab fruit pulp from Kenya and Mali had the highest moisture content at 9.82 g and 9.81 g 100^−1^ g, respectively, while that from Zambia had the lowest content (8.89 g 100^−1^ g) though not significantly (*p* < .05) different from Malawi. The moisture content of common fruits ranges between 75 and 95 g 100^−1^ g (Ruiz‐Rodríguez et al., 2011), in contrast, the pulp moisture content from all the baobab fruits irrespective of provenance ranged between 8.81 and 9.93 g 100^−1^ g (Table 3). It is worth mentioning that pulp moisture content was high in the East and West African provenances, but lowest in South African provenances (Table 3). This range in fruit pulp moisture content was found to be similar to the values previously reported in the literature (Lockett, Calvert, & Grivetti, 2000; Murray, Schoeninger, Bunn, Pickering, & Marlett, 2001). The pulp moisture content is low when compared to other commonly consumed African fruits, namely *Balanites aegyptiaca*,* S. birrea*,* T. indica*,* Dacryodes edulis*,* Vitex doniana*,* Uapaca kirkiana*,* Z. mauritiana*, and *Syzygium guineense*, some of which grow under the same environmental conditions as baobab (Stadlmayr et al., 2013). The low pulp moisture content recorded across all provenances in this study can be attributed to the low altitude, low annual precipitation, and moderately high temperatures as presented in Table 1. Other factors such as sunlight and wind exposure have been reported to contribute to fruit pulp dryness (Ruiz‐Rodríguez et al., 2011).

**Table 2:** Proximate composition of baobab fruit pulp

Country	Moisture (g 100 g^−1^)	Crude protein (g 100 g^−1^ dw)	Crude fiber (g 100 g^−1^ dw)	Ash (g 100 g^−1^ dw)
Tanzania	9.24 ± 0.281^b^	1.99 ± 0.132^b^	6.92 ± 0.737^b^	4.38 ± 0.125^a^
Zambia	8.89 ± 0.061^c^	2.24 ± 0.116^a^	7.09 ± 0.723^b^	4.34 ± 0.102^a^
Kenya	9.82 ± 0.028^a^	2.20 ± 0.220^a^	8.83 ± 2.564^a^	4.41 ± 0.108^a^
Malawi	8.94 ± 0.071^c^	1.91 ± 0.039^bc^	7.27 ± 0.083^b^	4.40 ± 0.054^a^
Mali	9.81 ± 0.050^a^	1.86 ± 0.077^c^	6.69 ± 0.706^b^	3.86 ± 0.531^b^
*p*	<.001	<.001	<.001	<.001
CV	1.7	6.2	15.6	7.1

Moisture based on 100 g fresh weight, all other parameters based on 100 g dry weight. For the same column, values (means ± *SEM*) followed by the same letter are not significantly different. CV, coefficient of variation.

**Table 3:** Baobab fruit pulp moisture, crude protein, crude fiber, and ash content according to the selected provenances

Provenance	Moisture (g 100 g^−1^)	Crude protein (g 100 g^−1^ dw)	Crude fiber (g 100 g^−1^ dw)	Ash (g 100 g^−1^ dw)
Kibwezi	9.79 ± 0.026^abc^	2.42 ± 0.051^b^	8.44 ± 1.937^ab^	4.23 ± 0.099^bc^
Taita	9.82 ± 0.032^ab^	2.08 ± 0.195^cd^	7.82 ± 0.828^abc^	4.66 ± 0.090^ab^
Malindi	9.85 ± 0.027^a^	2.04 ± 0.313^cdef^	9.61 ± 3.428^a^	4.48 ± 0.122^ab^
Chirundu	8.82 ± 0.063^e^	2.06 ± 0.158^cde^	7.10 ± 0.823^bc^	4.51 ± 0.130^ab^
Siavonga	8.95 ± 0.063^e^	2.65 ± 0.059^a^	7.75 ± 0.048^bc^	4.68 ± 0.084^a^
Mambwe	8.89 ± 0.054^e^	2.02 ± 0.111^cdef^	6.42 ± 0.943^c^	3.82 ± 0.085^c^
Kilolo	9.77 ± 0.436^abc^	2.44 ± 0.138^b^	6.80 ± 0.945^c^	4.40 ± 0.130^ab^
Kongwe	9.24 ± 0.267^bde^	1.58 ± 0.135^i^	6.84 ± 1.090^bc^	3.98 ± 0.155^c^
Moshi rural	8.98 ± 0.224^e^	1.82 ± 0.027^gh^	6.54 ± 0.083^c^	4.54 ± 0.088^a^
Mwanga	8.97 ± 0.063^e^	2.15 ± 0.179^c^	7.51 ± 0.291^bc^	4.59 ± 0.118^ab^
Mopti	9.57 ± 0.070^abcd^	1.97 ± 0.053^defg^	6.35 ± 0.940^c^	3.40 ± 0.586^d^
Sikasso	9.89 ± 0.016^a^	1.86 ± 0.033^dfgh^	6.63 ± 0.451^c^	3.97 ± 0.765^c^
Kayes	9.94 ± 0.038^a^	1.73 ± 0.090^hi^	6.52 ± 0.482^c^	3.86 ± 0.383^c^
Seguo	9.88 ± 0.057^a^	1.87 ± 0.108^dfgh^	7.25 ± 0.799^bc^	4.21 ± 0.181^bc^
Mangochi	8.94 ± 0.071^e^	1.91 ± 0.039^defg^	7.27 ± 0.083^bc^	4.40 ± 0.054^ab^
*p*	<.001	<.001	<.001	<.001
CV	4.0	6.2	15.6	7.1

Moisture based on 100 g fresh weight, all other parameters based on 100 g dry weight. For the same column, values (means ± *SEM*) followed by the same letter are not significantly different. CV, coefficient of variation.

The mean crude protein content ranged between 1.86 g 100 g^−1^ in Mali and 2.24 g 100 g^−1^ dw in Zambia, as shown in Table 2. At provenance level, Siavonga (Zambia) had the highest (2.65 g 100 g^−1^) mean pulp crude protein content, while Kongwe (Tanzania) and Kayes (Mali) had the lowest protein content at 1.57 g and 1.73 g 100 g^−1^ dw, respectively (Table 3). For crude protein content, there was no specific regional trend across the provenances. Previous studies conducted on baobab fruit pulp showed that pulp protein concentration ranged from 2.5 g to 3.6 g 100 g^−1^ dw (Assogbadjo et al., 2012; Lockett et al., 2000; Osman, 2004; Sidibé & Williams, 2002). Interestingly, the crude protein content of baobab pulp recorded in this study is comparable to that of *T. indica* fruit pulp, an indigenous fruit tree which grows under the same ecological conditions as baobab tree (De Caluwé, Halamová, & Van Damme, 2010). Other indigenous fruit trees, namely *S. birrea*,* Strychnos spinosa*, and *Vangueria infausta* growing in Africa were reported to have crude protein levels ranging from 1.3 to 3.7 g 100 g^−1^ (Amarteifio & Mosase, 2006). Even though baobab fruit pulp has a low protein content, it is much higher than that of commonly consumed fruits, namely oranges, mangoes, grapes, banana, and papaya which have a protein content of 0.7, 0.6, 0.5, 1.2, and 0.6 g 100 g^−1^, respectively (Rathore, 2009).

At the country level, mean pulp crude fiber content ranged from 6.69 g in Mali to 8.83 g 100 g^−1^ dw in Kenya as shown in Table 2. Malindi, Kibwezi, and Taita provenances (Kenya) had the highest levels of crude fiber content among all the study provenances. However, Taita provenance did not vary significantly (*p* < .05) from the other provenances in Tanzania, Zambia, Malawi, and Mali for crude fiber content (Table 3). This finding showed that the influence of environment on pulp crude fiber content was very minimal. Assogbadjo et al. (2012) reported that there were no significant differences among agro‐climatic zones of Benin for pulp fiber content. His finding concurs with our data which shows that the provenances in each country did not vary significantly (*p* < .05) for pulp fiber content. This study documents for the first time that the pulp fiber content is not influenced by the provenance of origin within each country, either in the west, east, or south of Africa. The fruit pulp fiber content reported in the literature are generally below 12.5 g 100 g^−1^ dw, with levels varying from 6.0 g (Osman, 2004) to 12.5 g 100 g^−1^ dw (Lockett et al., 2000; Sidibé & Williams, 2002). Other studies conducted on baobabs in Benin reported a mean of 11.0 g 100 g^−1^ dw for pulp crude fiber for each of the three agro‐climatic zones, namely Sudanian, the Sudano‐Guinean zone, and the Guineo‐Congolian zone (Assogbadjo et al., 2012). Based on the findings of the present study, the fruit pulp can be recommended as a source of dietary fiber.

The pulp ash content was highest in Kenya (4.41 g 100 g^−1^ dw) and the lowest in Mali (3.86 g 100 g^−1^ dw) as shown in Table 2. Among the study provenances, Siavonga had the highest mean ash content though it was not significantly different (*p *<* *.05) from Taita, Mwanga, Moshi, Chirundu, Malindi, Mangochi, and Kilolo, as presented in Table 3. Mopti in Mali had the lowest mean ash content at 3.40 g 100 g^−1^ dw. The average pulp ash content found in this study for Mali concurs with the range 3.68–4.78 g 100 g^−1^ dw reported for baobabs in Mali, Burkina Faso, and Niger by Parkouda et al. (2012). Since the ash content is a reflection of the inorganic matter of the pulp tissue, our findings would suggest that baobabs from east and southern Africa provenances are rich in mineral elements, while those from West Africa (Mali) are poor sources of such elements. Moreover, the West African provenances experienced warmer temperatures than the other regions (Table 1), and this observation could have influenced the pulp biomass.

A comparative assessment was conducted for baobab seeds proximate composition for five countries. The results (Table 4) showed that there were statistically significant differences (*p *<* *.001) among the countries for the seed proximate composition. The highest (7.01 g 100^−1 ^g) seed moisture content was recorded in Kenya, while the lowest (5.67 g 100^−1 ^g) was found in seeds from Mali. For seed crude protein content, the levels ranged from 12.69 g in Mali to 15.18 g 100 g^−1^ dw in Malawi. At provenance level, Malindi had the highest (7.04 g 100^−1 ^g) seed moisture content, though it was not significantly (*p *<* *.05) different from Taita, Kibwezi, Kongwe, Moshi, and Kilolo (Table 5). Mean crude protein content ranged between 11.21 g (Seguo) and 16.02 g 100^−1 ^g dw (Kilolo). Overall, East African provenances had much higher protein content than the West African provenances as presented in Table 5. The seed moisture, ash, and macronutrients were present in the following order: carbohydrates > crude fibers > crude protein > crude fat > moisture > ash.

**Table 4:** Proximate composition of baobab seeds

Country	Moisture (g 100 g^−1^)	Protein (g 100 g^−1^ dw)	Fiber (g 100 g^−1^ dw)	Fat (g 100 g^−1^ dw)	Carbohydrates (g 100 g^−1^ dw)	Ash (g 100 g^−1^ dw)
Zimbabwe	5.88 ± 0.202^d^	14.45 ± 0.632^a^	22.18 ± 0.712^c^	12.18 ± 0.562^ab^	64.01 ± 1.104^b^	3.48 ± 0.263^e^
Tanzania	6.65 ± 0.137^b^	14.92 ± 0.729^a^	23.83 ± 0.584^a^	12.60 ± 0.671^a^	61.75 ± 0.916^c^	4.08 ± 0.158^ac^
Malawi	5.90 ± 0.118^d^	15.18 ± 0.391^a^	23.56 ± 0.136^ab^	12.66 ± 0.510^a^	62.08 ± 0.811^c^	4.18 ± 0.109^a^
Zambia	6.34 ± 0.137^c^	13.77 ± 0.892^b^	22.93 ± 0.313^b^	11.37 ± 0.998^c^	64.50 ± 1.410^b^	4.03 ± 0.093^cd^
Kenya	7.01 ± 0.067^a^	14.43 ± 0.365^a^	23.31 ± 2.839^ab^	12.41 ± 0.505^a^	61.94 ± 0.701^c^	3.92 ± 0.193^d^
Mali	5.67 ± 0.093^e^	12.69 ± 0.793^c^	23.74 ± 0.277^a^	11.79 ± 0.759^bc^	65.69 ± 0.974^a^	4.17 ± 0.160^ab^
*p*	<.001	<.001	<.001	<.001	<.001	<.001
CV	1.8	5.5	4.3	6.6	3.7	1.8

For the same column, values (means ± *SEM*) followed by the same letter are not significantly different. CV, coefficient of variation.

**Table 5:** Proximate composition of baobab seeds according to the study provenances

Provenance	Moisture (g 100 g^−1^)	Protein (g 100 g^−1^ dw)	Fiber (g 100 g^−1^ dw)	Fat (g 100 g^−1^ dw)	Carbohydrates (g 100 g^−1^ dw)	Ash (g 100 g^−1^ dw)
Kibwezi	6.97 ± 0.043^abc^	14.99 ± 0.466^ab^	22.47 ± 1.310^efg^	12.62 ± 0.326^abc^	61.11 ± 0.857^fg^	4.01 ± 0.290^ab^
Taita	7.01 ± 0.056^ab^	15.93 ± 0.300^a^	20.93 ± 3.364^g^	13.49 ± 0.350^a^	59.50 ± 0.574^g^	3.78 ± 0.051^ab^
Malindi	7.04 ± 0.088^a^	13.26 ± 0.258^d^	25.11 ± 3.620^ab^	11.77 ± 0.677^abcd^	63.74 ± 0.561^cde^	3.89 ± 0.062^ab^
Chirundu	6.31 ± 0.145^bdef^	13.46 ± 1.045^d^	22.90 ± 0.447^defg^	11.21 ± 0.713^acd^	64.91 ± 1.304^bcd^	4.10 ± 0.080^ab^
Siavonga	6.50 ± 0.156^abcde^	13.85 ± 0.535^bd^	23.58 ± 0.273^cde^	10.35 ± 1.354^d^	65.16 ± 1.426^bc^	4.14 ± 0.087^ab^
Mambwe	6.20 ± 0.104^befg^	14.00 ± 1.000^bcd^	22.32 ± 0.139^efg^	12.53 ± 0.805^abc^	63.43 ± 1.495^de^	3.83 ± 0.108^b^
Kilolo	6.61 ± 0.175^abcde^	16.02 ± 0.895^a^	23.29 ± 0.175^cde^	13.04 ± 0.264^abc^	60.03 ± 0.935^g^	4.31 ± 0.082^ab^
Kongwe	6.95 ± 0.175^abc^	15.10 ± 0.568^a^	22.31 ± 0.513^efg^	13.35 ± 0.209^ab^	60.50 ± 0.647^g^	4.09 ± 0.108^ab^
Moshi	6.81 ± 0.090^abcd^	13.17 ± 0.733^d^	26.25 ± 0.960^a^	11.93 ± 0.354^abcd^	64.23 ± 0.649^bcd^	3.86 ± 0.163^b^
Mwanga	6.26 ± 0.076^bef^	15.38 ± 0.681^a^	23.47 ± 0.386^cde^	12.08 ± 1.249^abcd^	62.22 ± 1.282^ef^	4.06 ± 0.234^a^
Mopti	5.51 ± 0.151^h^	13.09 ± 0.338^d^	23.99 ± 0.226^bcd^	11.43 ± 0.303^abcd^	65.82 ± 0.547^b^	4.14 ± 0.124^ab^
Sikasso	5.86 ± 0.058^fgh^	12.93 ± 0.668^d^	23.20 ± 0.212^def^	11.98 ± 0.268^abcd^	64.83 ± 0.681^bcd^	4.39 ± 0.140^a^
Kayes	5.58 ± 0.079^h^	13.66 ± 1.034^d^	23.05 ± 0.306^defg^	12.36 ± 0.519^abcd^	64.50 ± 0.890^bcd^	3.89 ± 0.201^ab^
Seguo	5.71 ± 0.038^gh^	11.21 ± 0.973^e^	24.63 ± 0.346^bc^	11.45 ± 1.352^abcd^	67.44 ± 1.490^a^	4.19 ± 0.171^ab^
FRC	5.92 ± 0.144^fgh^	14.98 ± 0.423^abc^	22.55 ± 0.598^efg^	12.88 ± 0.236^abc^	62.26 ± 0.596^ef^	3.96 ± 0.209^ab^
Hwange	5.85 ± 0.140^fgh^	13.92 ± 0.469^bcd^	21.81 ± 0.385^fg^	11.48 ± 0.510^abcd^	65.76 ± 0.929^b^	3.00 ± 0.157^c^
Mangochi	5.90 ± 0.118^fgh^	15.18 ± 0.391^a^	23.56 ± 0.136^cde^	12.66 ± 0.510^efgh^	62.08 ± 0.811^cde^	4.18 ± 0.109^ab^
*p*	<.001	<.001	<.001	<.001	<.001	<.001
CV	6.6	5.8	4.5	12.2	1.9	9.6

For the same column, values (means ± *SEM*) followed by the same letter are not significantly different. CV, coefficient of variation.

The very low seed moisture content recorded for Malian provenances can be correlated with the region's high temperatures (Table 1) which could contribute to seed desiccation. The differences in seed moisture levels across the study countries could best be attributed to the area's temperature. The seed protein values in this study ranged from 13.00 to 16.50 g 100 g^−1^ dw, which is within the range reported in previous studies conducted in West Africa (Parkouda et al., 2012).

Seed crude protein levels reported here are twice lower than those reported for baobab seeds in Benin by Assogbadjo et al. (2012). The results (Table 5) show a regional trend in seed protein content where seeds from West African provenances had the lowest crude protein content, while those from East African provenances had the highest content. This would suggest that other than the warm climate of Mali, the region has soils that are poor in nitrogen, nitrates, or nitrites which make up proteins in plant tissues. The high seed protein content is a reflection of a high amino acid content within the seed tissue. Therefore, seeds from these provenances/countries could be used to process flours rich in proteins and their subsequent consumption in local diets could be a possible nutritional strategy to augment cereal diets low in proteins. Baobab seeds have been documented to contain high amounts of essential amino acids such as lysine and tryptophan (Glew et al., 1997; Osman, 2004). Because lysine is found in very minimal quantities in most cereal plants, baobab seed protein could potentially be used to improve cereal protein quality, especially in areas where people are dependent on cereal‐dominated diets (Osman, 2004). This finding provides a strong basis for the recommendation of baobab seeds as a source of protein in protein‐poor diets of baobab‐growing areas and the potential utilization of the seed flour in fortifying other foods.

The whole seeds of *A. digitata* analyzed in our study contain high levels of crude fiber content (20.0–26.5 g 100 g^−1^ dw), as shown in Table 5. However, values three times lower than those reported here were recorded in separate studies conducted in Nigeria and Benin (6–8 g 100 g^−1^ dw) (Assogbadjo et al., 2012; Nkafamiya, Osemeahon, Dahiru, & Umaru, 2007). In other studies, Lockett et al. (2000) reported much higher crude fiber content of 47.6 g 100 g^−1^ dw. The differences recorded in our study for seed fiber content among the provenances suggest that the geographical conditions significantly influenced the concentration of fiber in the tissue. However, there was no specific regional trend for seed fiber content. The values reported here for seed fat content concur with those previously reported for baobabs (Glew et al., 1997; Nkafamiya et al., 2007; Osman, 2004; Parkouda et al., 2012, 2015). However, higher values for crude fat have been reported. For instance, the crude fat concentration of 28 g 100 g^−1^ on average was reported in three provenances in Benin (Assogbadjo et al., 2012). These differences in the seed fat content among the study provenances can be attributed more to genetic factors than geographical conditions since variations were very minimal across the provenances.

The ash content of 3.0–4.5 g 100 g^−1^ dw in this study was found to concur with previously reported values for baobab seeds (Nkafamiya et al., 2007; Osman, 2004; Parkouda et al., 2012). The crude fat and ash content of baobab are comparable to that of *Neonotonia wightii* (Wight & Arn.) Lackey (Fabaceae) seeds, used as food by Malayali tribals of India (Viswanathan, Thangadurai, & Ramesh, 2001). Baobab seeds are eaten raw, ground into powder or are roasted, and have a pleasant nutty flavor (Chadare et al., 2009). The seed flour could serve as an important source of energy and protein for local populations, especially in East Africa where the seeds are hardly included in the diet. In the Sahel region of Africa, the seeds are used in the preparation of local sauces as thickening agents after pounding or as flavor enhancers when fermented (Diop, Sakho, Dornier, Cisse, & Reynes, 2005). One of the numerous products of baobab seeds is *Maari*, a condiment obtained from fermenting baobab seeds in Burkina Faso, though it is found under other several names in Mali and Nigeria among other West African countries where it is part of the daily diet (Parkouda et al., 2015). *Maari* is known in Nigeria as *Dadawa*,* Higgi*, or *Issai* and in Benin as *Dikouanyouri* (Chadare et al., 2009; Nkafamiya et al., 2007). Since the fermentation of baobab seeds induces several biochemical changes which decrease protein and carbohydrate but increase fatty acids and free amino acids (Parkouda et al., 2015), this preparation method should be adopted by people in the East and Southern Africa. It is estimated that consumption of 20 g of the seed flour can cover 15%–34% of the protein RDI for children, while for pregnant women 60 g can cover 27% of the RDI based on the highest reported content (Chadare et al., 2009).

Moreover, consumption of 100 g can cover 22% of the energy RDI for pregnant women and 29.4% of energy RDI for children (Chadare et al., 2009). It is important to note that such calculations are usually done considering the digestibility and bioavailability of the nutrients. Baobab seeds are hardly consumed as a food supplement in East Africa (Gebauer et al., 2016), the findings from this study provide evidence that the seeds are nutritious and should be included in our diets. More often the seeds with pulp on them are roasted and sold as “mabuyu” (Gebauer et al., 2016), but people hardly crack the seeds to extract and eat the fat‐rich kernel. Furthermore, stakeholders should adopt nutritional policies that promote the consumption of roasted seeds or seed flour among school‐going children and communities facing malnutrition.

### 3.2 Mineral element concentration based on the region of origin

The mineral composition—macroelements (Ca, K, Mg, Na, and P) and microelements (Fe, Cu, Mn, and Zn)—of baobab fruit pulp from the different countries are presented in Table 6. Among the macroelements, potassium, calcium, and magnesium were the most abundant, while sodium and phosphorus were the least abundant in the pulp tissue. From the results, there was significant variation (*p *<* *.001) among the five countries for the amount of all the analyzed elements. At provenance level (Table 7), the mean pulp calcium levels ranged from 1,800 mg kg^−1^ in Mopti (Mali) to 4,300 mg kg^−1^ in Mangochi (Malawi). The highest pulp potassium content (25.3 mg g^−1^) was recorded in Malindi (Kenya), while the lowest level was recorded in three provenances, Mali (Kayes (13.6 μg g^−1^), Sikasso (13.3 μg g^−1^), and Mopti (13.8 μg g^−1^). Pulp iron was highest in Mwanga and Kibwezi at 64.15 μg g^−1^ and 61.50 μg g^−1^, respectively, being almost fivefold the content reported in any of the Malian provenances which ranged between 12.07 μg g^−1^ and 14.75 μg g^−1^.

**Table 6:** Mineral concentration of baobab fruit pulp from different countries

Country	Ca (mgkg^−1^)	K (mg g^−1^)	Mg (mg kg^−1^)	Na (mg kg^−1^)	P (mg kg^−1^)	Cu (μg g^−1^)	Fe (μg g^−1^)	Mn (μg g^−1^)	Zn (μg g^−1^)
Tanzania	3,200 ± 100^b^	20.3 ± 0.61^c^	1,600 ± 100^b^	600 ± 70^b^	700 ± 50^d^	47.7 ± 2.4^b^	34.0 ± 1.5^c^	11.9 ± 0.9^d^	38.6 ± 3.9^c^
Zambia	3,200 ± 110^b^	20.0 ± 0.71^c^	1,600 ± 60^b^	400 ± 150^c^	800 ± 30^c^	42.9 ± 0.8^d^	48.3 ± 1.6^b^	19.4 ± 0.6^b^	41.3 ± 2.2^b^
Kenya	2,700 ± 100^c^	21.2 ± 0.76^b^	1,300 ± 50^c^	500 ± 170^b^	600 ± 20^e^	45.4 ± 0.8^c^	57.4 ± 2.0^a^	27.2 ± 0.5^a^	71.6 ± 2.3^a^
Malawi	4,300 ± 120^a^	22.2 ± 0.64^a^	2,300 ± 60^a^	1,000 ± 30^a^	1,100 ± 30^a^	28.9 ± 2.4^e^	26.7 ± 2.2^d^	16.3 ± 0.8^c^	22.5 ± 2.0^e^
Mali	2,500 ± 190^d^	14.1 ± 0.81^d^	1,600 ± 70^b^	500 ± 70^b^	820 ± 50^b^	53.1 ± 5.1^a^	13.1 ± 3.3^e^	8.6 ± 1.9^e^	27.5 ± 3.6^d^
*p*	<.001	<.001	<.001	<.001	<.001	<.001	<.001	<.001	<.001
CV	4.4	3.8	4.6	19.1	5.3	6.7	6.8	8.0	8.1

For the same column, values (means ± *SEM*) followed by the same letter are not significantly different. CV, coefficient of variation.

**Table 7:** Baobab fruit pulp mineral element composition according to the selected provenances

Provenance	Ca (mg kg^−1^)	K (mg g^−1^)	Mg (mg kg^−1^)	Na (mg kg^−1^)	P (mg kg^−1^)	Cu (μg g^−1^)	Fe (μg g^−1^)	Mn (μg g^−1^)	Zn (μg g^−1^)
Kibwezi	2,800 ± 100^e^	18.5 ± 0.05^fg^	1,300 ± 50^fg^	300 ± 100^efg^	570 ± 20^e^	47.1 ± 0.80^def^	61.5 ± 2.14^ab^	26.3 ± 0.48^b^	81.3 ± 2.49^a^
Taita	2,400 ± 80^fg^	17.6 ± 0.62^gh^	1,600 ± 50^de^	300 ± 70^efg^	750 ± 30^cd^	49.1 ± 0.86^cde^	58.0 ± 2.23^b^	23.8 ± 0.43^a^	79.1 ± 2.08^a^
Malindi	2,900 ± 100^de^	25.3 ± 0.89^a^	1,200 ± 40^g^	700 ± 230^bc^	570 ± 20^e^	42.3 ± 0.75^fgh^	53.1 ± 1.84^c^	29.5 ± 0.56^cd^	58.9 ± 2.10^c^
Chirundu	3,000 ± 110^d^	21.1 ± 0.74^de^	1,700 ± 60^cd^	700 ± 210^bc^	850 ± 30^b^	45.1 ± 0.82^defg^	40.7 ± 1.48^e^	11.3 ± 0.20^g^	16.6 ± 0.57^h^
Siavonga	3,600 ± 130^bc^	17.2 ± 0.61^h^	1,900 ± 70^b^	200 ± 80^fg^	730 ± 30^d^	43.8 ± 0.76^efgh^	45.7 ± 1.30^d^	23.6 ± 0.97^d^	66.2 ± 3.38^b^
Mambwe	2,900 ± 100^de^	21.9 ± 0.77^cd^	1,200 ± 40^g^	400 ± 110^ef^	780 ± 30^cd^	39.9 ± 0.83^h^	58.5 ± 2.05^b^	23.4 ± 0.42^d^	41.2 ± 1.50^d^
Kilolo	3,600 ± 100^bc^	19.3 ± 0.55^f^	1,200 ± 40^fg^	700 ± 80^bc^	770 ± 50^cd^	46.8 ± 2.74^def^	16.5 ± 0.96^g^	3.7 ± 0.53^h^	26.0 ± 1.46^fg^
Kongwe	3,500 ± 130^bc^	18.7 ± 0.54^fg^	1,800 ± 60^c^	800 ± 80^b^	740 ± 50^d^	49.1 ± 3.66^d^	17.6 ± 1.22^g^	5.0 ± 1.55^h^	26.0 ± 7.09^fg^
Moshi R	3,400 ± 90^c^	20.3 ± 0.46^e^	1,900 ± 180^b^	600 ± 40^c^	800 ± 50^bc^	53.6 ± 1.46^bc^	38.0 ± 1.07^e^	13.8 ± 0.70^f^	39.2 ± 1.67^de^
Mwanga	2,200 ± 80^g^	23.1 ± 0.82^b^	1,300 ± 50^f^	200 ± 70^g^	570 ± 20^e^	41.5 ± 0.72^gh^	64.1 ± 2.23^a^	25.2 ± 0.46^bc^	63.1 ± 2.17^bc^
Mopti	1,800 ± 70^h^	13.8 ± 0.40^j^	1,200 ± 30^fg^	400 ± 8^ef^	840 ± 40^b^	56.5 ± 3.31^ab^	12.8 ± 0.84^h^	4.4 ± 0.37^h^	23.5 ± 1.33^g^
Sikasso	2,400 ± 80^f^	13.3 ± 0.40^j^	1,500 ± 50^e^	600 ± 80^cd^	850 ± 30^b^	44.8 ± 2.64^efg^	13.1 ± 0.85^h^	3.8 ± 0.53^h^	22.3 ± 1.31^g^
Kayes	3,000 ± 360^de^	13.7 ± 1.53^j^	1,900 ± 100^b^	600 ± 90^cd^	760 ± 90^cd^	58.7 ± 9.70^a^	14.7 ± 6.92^gh^	14.0 ± 3.84^f^	35.5 ± 7.24^e^
Seguo	2,900 ± 90^de^	15.6 ± 0.50^i^	1,700 ± 70^cd^	600 ± 20^cd^	840± 40^b^	53.3 ± 1.53^bc^	12.0 ± 0.59^h^	13.0 ± 0.59^f^	30.0 ± 0.84^f^
Mangochi	4,300 ± 120^a^	22.2 ± 0.64^bc^	2,300 ± 60^a^	1,000 ± 30^a^	1,100 ± 30^a^	28.9 ± 2.40^i^	26.7 ± 2.20^f^	16.3 ± 0.80^e^	22.5 ± 2.00^g^
*p*	<.001	<.001	<.001	<.001	<.001	<.001	<.001	<.001	<.001
CV	4.4	3.8	4.6	19.1	5.3	6.7	6.8	8.0	8.1

For the same column, values (means ± *SEM*) followed by the same letter are not significantly different. CV, coefficient of variation.

At the country level, Kenya had the highest mean iron (57.4 μg g^−1^), manganese (27.2 μg g^−1^), and zinc (71.6 μg g^−1^) content, while Mali had the lowest mean calcium (2,500 mg kg^−1^), potassium (14.1 mg g^−1^), iron (13.1 μg g^−1^), and manganese (8.6 μg g^−1^) levels. Even though Mali had the lowest concentration for most of the pulp elements, it had the highest (53.1 μg g^−1^) mean for pulp copper content. According to Table 6, and with reference to most of the countries, the elements occurred in the following order: K > Ca > Mg > P > Na > Cu > Fe > Zn > Mn, although this pattern was challenged in some countries due to variation in micronutrient levels. There was low variability (CV = 3%–8%) among the countries in the macro‐ and microelements concentration but with the exception of sodium (CV = 19.1%).

The pulp calcium concentration recorded here for Malian provenances averaging between 1,800 mg and 3,000 mg kg^−1^ was found to agree with the range (1,600–3,090 mg kg^−1^) previously reported in Mali by Simbo et al. (2013). The regional variation in pulp calcium content can be linked to the area's geographical conditions. Pulp from Malawi had remarkably high calcium content which can be used to supplement diets poor in calcium, more so for children, pregnant and lactating mothers, and the elderly. Inadequate dietary supply and a high risk of calcium deficiency in Malawi have been previously reported based on food balance sheets (FBSs) (Broadley et al., 2012; Joy et al., 2014). Interestingly, the calcium content reported here for baobab is more than that of commonly consumed fruits such as guava (18 mg 100 g^−1^), orange (11 mg 100 g^−1^), pear (4 mg 100 g^−1^), and strawberry (22 mg 100 g^−1^) as documented by Mahapatra, Mishra, Basak, and Panda (2012). Calcium is an important element in bone development, and a high intake is recommended especially for pregnant women and young children (Insel, Ross, McMahon, & Bernstein, 2011). Even though dairy products are a major source of calcium for human health, this study reveals that baobab fruit pulp is an excellent source of dietary calcium for people inhabiting the dryer regions of Africa where dairy farming is a challenge, but the growth of baobab is evident.

Potassium is the most abundant mineral element of the fruit pulp. Pulp potassium, magnesium, sodium, and phosphorus levels in this study ranged between 14.1–22.2 mg g^−1^, 1,300–2,300 mg kg^−1^, 400–1,000 mg kg^−1^, and 600–1,100 mg kg^−1^, respectively. The high potassium content recorded in Malindi as opposed to other provenances can be correlated with the area's altitude. According to Table 1, Malindi is located at a very low altitude (8 m), near the Indian Ocean, and with high rainfall which could influence soil potassium mobility. The potassium content of baobab pulp is much higher than that found in commonly consumed fruits, such as guava (4.17 mg g^−1^), oranges (2.00 mg g^−1^), apples (0.90 mg g^−1^), and bananas (3.58 mg g^−1^) (Mahapatra et al., 2012). Potassium and sodium are known to regulate the muscle contraction and nerve impulse transmission, and a high K/Na ratio could play a role in excretion of excess water and salt (Arthey & Ashurst, 2001). In this study, the pulp sodium content was lower than potassium suggesting a high K/Na ratio.

Baobab fruit pulp from Malawi had the highest concentration of potassium, magnesium, sodium, and phosphorus which forms a firm basis for the recommendation of pulp from Malawi as an excellent source of macroelements. The high content of these elements in the pulp tissue could be attributed to soil properties other than the climatic conditions. Magnesium and phosphorus are also important for growth and healthy maintenance of bones, teeth, and muscles (Insel et al., 2011).

The pulp copper, iron, manganese, and zinc concentrations reported in this study show that there is value in consuming baobab fruit pulp when available. In literature, microelement concentration of baobab pulp is reported to vary greatly from one author to the other due to different methods of sample preparation and analytical methods (Chadare et al., 2009). The present study avoided such discrepancies by using the same method to document for the first time the elemental composition of baobab parts across east, west, and southern African regions. Regional variation was noted for pulp microelement concentration. This variability across the provenances could be regional precipitation which influences soil mineral element mobility and adsorption by plants. Moreover, soil physicochemical properties and agricultural activities could also influence the pulp microelement content.

The mean estimated risk of iron and zinc deficiency in eastern region of Africa is 14% and 75%, respectively (Joy et al., 2014). Since baobab pulp from Kenya is reported to be rich in iron and zinc, it could be used as a potential dietary supplement in East Africa and regions in the Western world that face iron and zinc deficiency. The findings of this study would support the recommendation of fruit pulp as a possible strategy to tackle the problem of macro‐ and microelement deficiencies through its inclusion in foods during fortification. In sub‐Saharan Africa (SSA), nutrition‐related programs for school‐going children should consider the provision of pulp juices and also porridges/gruels that contain baobab pulp as an ingredient. The results of this study suggest that baobab fruit pulp is a good source of macro‐ (calcium, potassium, and magnesium) and microelements (iron, manganese, and zinc), but the levels are influenced by the provenances of origin.

Table 8 presents the elemental composition of baobab seeds across different study countries. Among the macroelements, potassium was the most abundant element followed by phosphorus, magnesium, and calcium. Regarding the microelements, iron and copper were the most abundant. From the analysis conducted, there were statistically significant differences (*p *<* *.001) among the countries for all the investigated elements. The mean seed calcium levels ranged from 2000 mg kg^−1^ in Kenya to 3,200 mg kg^−1^ in Malawi. Malawi had the highest magnesium and sodium content, while Zimbabwe had the lowest magnesium, phosphorus, and zinc content (Table 5). Zambia showed the highest seed phosphorus and copper concentration, while Kenya had the lowest seed copper content. Seed iron levels in Kenya (63.7 μg g^−1^) were two to three times more than those found in Mali (25.8 μg g^−1^). At provenance level, Moshi and Kilolo had the highest seed potassium level at 14.0 mg g^−1^ and 13.4 mg g^−1^, respectively, though not significantly different from each other, while Seguo had the lowest content at 6.4 mg g^−1^. Seed copper content was very low in East African provenances, but high in southern and western African provenances, as shown in Table 9. The data in Table 8 show that the macro‐ and microelements accumulation at country level was K > P > Mg > Ca > Na > Fe > Cu > Zn > Mn. The countries did not show large variability (CV = 3%–5%) for most of the selected macro‐ and microelements except for potassium and sodium, 12.3% and 14.6%, respectively. Generally, baobab seeds are a rich source of nutritionally essential minerals, particularly potassium, phosphorus, magnesium, and calcium, which are the most abundant elements as evidenced by our results.

**Table 8:** Mineral concentration of baobab seeds according to the different countries

Country	Ca (mg kg^−1^)	K (mg g^−1^)	Mg (mg kg^−1^)	Na (mg kg^−1^)	P (mg g^−1^)	Cu (μg g^−1^)	Fe (μg g^−1^)	Mn (μg g^−1^)	Zn (μg g^−1^)
Zimbabwe	2,300 ± 160^e^	8.8 ± 0.22^c^	3,100 ± 100^f^	200 ± 20^d^	5.5 ± 0.3^d^	32.5 ± 3.2^c^	58.3 ± 4.9^b^	19.9 ± 0.9^c^	18.9 ± 3.8^e^
Tanzania	2,900 ± 90^b^	12.0 ± 0.35^a^	4,000 ± 120^b^	400 ± 40^b^	6.3 ± 0.4^b^	28.8 ± 2.0^d^	44.2 ± 3.1^e^	10.5 ± 0.6^d^	26.9 ± 2.2^c^
Malawi	3,200 ± 90^a^	10.6 ± 0.28^b^	4,200 ± 120^a^	600 ± 20^a^	5.9 ± 0.2^c^	30.0 ± 3.1^d^	46.5 ± 3.8^d^	8.9 ± 0.8^e^	33.3 ± 2.6^a^
Zambia	2,400 ± 90^d^	10.4 ± 0.36^b^	3,600 ± 220^d^	200 ± 60^d^	7.7 ± 0.3^a^	46.3 ± 0.8^a^	51.0 ± 1.8^c^	22.4 ± 0.4^b^	31.9 ± 1.1^ab^
Kenya	2,000 ± 70^f^	9.1 ± 0.31^c^	3,300 ± 120^e^	190 ± 60^e^	6.0 ± 0.2^c^	27.5 ± 0.6^e^	63.7 ± 2.3^a^	20.1 ± 0.6^c^	21.8 ± 0.8^d^
Mali	2,600 ± 80^c^	9.0 ± 0.24^c^	3,800 ± 190^c^	300 ± 10^c^	6.1 ± 0.2^c^	41.0 ± 2.1^b^	25.8 ± 1.4^f^	23.8 ± 1.3^a^	31.3 ± 1.5^b^
*p*	<.001	<.001	<.001	<.001	<.001	<.001	<.001	<.001	<.001
CV	3.2	12.3	4.4	14.6	4.4	4.8	5.2	4.3	5.9

For the same column, values (means ± *SEM*) followed by the same letter are not significantly different CV, coefficient of variation..

**Table 9:** Mineral element content of baobab seeds according to the selected provenances

Provenance	Ca (mg kg^−1^)	K (mg g^−1^)	Mg (mg kg^−1^)	Na (mg kg^−1^)	P (mg kg^−1^)	Cu (μg g^−1^)	Fe (μg g^−1^)	Mn (μg g^−1^)	Zn (μg g^−1^)
Kibwezi	1,900 ± 60^j^	9.1 ± 0.29^def^	3,320 ± 120^gij^	170 ± 50^h^	5.6 ± 0.19^i^	28.2 ± 0.775^ij^	70.0 ± 2.598^a^	19.3 ± 0.734^e^	23.2 ± 0.811^i^
Taita	1,800 ± 60^j^	9.7 ± 0.34^bcdef^	3,580 ± 130^efg^	190 ± 60^gh^	6.2 ± 0.21^def^	30.3 ± 0.533^hi^	67.3 ± 2.397^a^	15.2 ± 0.275^f^	36.1 ± 1.392^bc^
Malindi	2,350 ± 80^gh^	8.9 ± 0.31^def^	3,240 ± 110^j^	200 ± 60^gh^	6.5 ± 0.23^cde^	25.7 ± 0.452^jk^	56.0 ± 2.003^cd^	23.1 ± 0.434^bc^	14.6 ± 0.284^k^
Chirundu	2,340 ± 80^ghi^	10.4 ± 0.36^bcde^	3,570 ± 138^fgh^	200 ± 60^gh^	7.5 ± 0.27^b^	41.9 ± 0.748^c^	48.1 ± 1.671^fg^	23.1 ± 0.439^bc^	28.7 ± 0.980^efg^
Siavonga	2,600 ± 100^ef^	10.7 ± 0.37^bcd^	3,900 ± 340^de^	220 ± 70^gh^	8.2 ± 0.32^a^	49.6 ± 0.870^b^	51.6 ± 1.797^ef^	24.2 ± 0.445^ab^	36.1 ± 1.226^b^
Mambwe	2,500 ± 90^f^	10.3 ± 0.36^bcdef^	3,450 ± 120^ghij^	240 ± 70^fgh^	7.6 ± 0.26^b^	47.4 ± 0.837^b^	53.5 ± 1.863^de^	19.9 ± 0.363^de^	30.9 ± 1.077^de^
Kilolo	3,500 ± 100^a^	13.4 ± 0.36^a^	4,200 ± 120^bc^	450 ± 10^cd^	6.8 ± 0.19^c^	24.5 ± 2.070^kl^	36.6 ± 3.059^h^	7.4 ± 0.655^h^	28.1 ± 2.449^fgh^
Kongwe	3,500 ± 100^a^	11.6 ± 0.33^b^	4,700 ± 130^a^	540 ± 20^b^	6.6 ± 0.74^cd^	32.1 ± 2.828^gh^	47.7 ± 4.080^g^	7.8 ± 0.655^gh^	33.0 ± 2.876^cd^
Moshi R	2,900 ± 80^c^	14.0 ± 0.39^a^	4,000 ± 110^cd^	470 ± 10^c^	6.1 ± 0.18^efgh^	22.9 ± 1.946^l^	32.3 ± 2.739^i^	7.6 ± 0.707^h^	26.8 ± 2.449^gh^
Mwanga	1,900 ± 70^j^	0.90 ± 0.31^d^	3,400 ± 120^ghij^	250 ± 80^fg^	5.7 ± 0.20^fhi^	35.7 ± 0.652^ef^	60.0 ± 2.272^bc^	19.3 ± 0.352^e^	19.9 ± 0.449^j^
Mopti	2,400 ± 70^g^	10.9 ± 0.32^ef^	3,800 ± 110^de^	390 ± 10^de^	5.9 ± 0.17^fghi^	37.0 ± 2.070^de^	24.4 ± 1.604^j^	23.0 ± 1.414^c^	26.2 ± 1.535^h^
Sikasso	2,680 ± 80^de^	9.4 ± 0.27^cdef^	4,000 ± 330^cd^	380 ± 10^e^	6.8 ± 0.20^c^	35.4 ± 2.035^ef^	24.2 ± 1.711^j^	24.6 ± 1.336^a^	26.0 ± 1.389^h^
Kayes	2,720 ± 80^d^	9.6 ± 0.27^cdef^	3,700 ± 110^ef^	400 ± 10^cde^	5.5 ± 0.16^i^	38.5 ± 2.255^d^	17.9 ± 0.866^k^	23.5 ± 1.291^abc^	30.1 ± 1.683^ef^
Seguo	2,600 ± 80^ef^	6.5 ± 4.76^g^	3,500 ± 100^ghi^	300 ± 10^f^	6.1 ± 0.20^efg^	52.7 ± 1.946^a^	35.7 ± 1.254^hi^	24.0 ± 0.926^abc^	42.6 ± 1.581^a^
FRC	2,500 ± 100f	9.0 ± 0.17^def^	3,300 ± 70^gij^	220 ± 10^gh^	6.1 ± 0.21^efg^	33.9 ± 2.791^fg^	56.4 ± 1.80^cd^	19.2 ± 0.585^e^	22.6 ± 3.64^i^
Hwange	2,200 ± 120^h^	8.6 ± 0.14^f^	2,900 ± 60^k^	250 ± 10 ^f^	5.0 ± 0.09^j^	31.1 ± 1.443^h^	60.3 ± 4.63^b^	20.5 ± 0.618^d^	15.2 ± 1.05^k^
*p*	<.001	<.001	<.001	<.001	<.001	<.001	<.001	<.001	<.001
CV	3.3	12.3	4.4	14.6	4.4	4.9	6.2	4.5	6.1

For the same column, values (means ± *SEM*) followed by the same letter are not significantly different. CV, coefficient of variation.

In most of the provenances, the potassium levels were similar to previously reported values by Osman (2004), but slightly lower than the average content (13.0–16.5 mg g^−1^) reported for different agro‐climatic zones in Benin (Assogbadjo et al., 2012). Regional variation was noted for seed potassium content whereby Tanzania provenances had the highest levels (Table 9). This observation can be correlated with soil characteristics other than the geographical conditions since other provenances in our study had similar environmental conditions but had low seed potassium. The seed magnesium content which ranged between 2,900 and 4,700 mg kg^−1^ among the different provenances was very similar to the range mentioned for the Benin provenances. The low seed calcium levels reported in Mwanga, Kibwezi, and Taita provenances in East Africa can be explained by geographical conditions shown in Table 1 since these regions experience almost the same annual temperature and rainfall. Moreover, the soil characteristics in these provenances could potentially influence the seed calcium content, an assumption that would need to be researched.

For most of the elements investigated in our study, the levels vary between the provenances in each country. This clearly indicates that elemental composition of the fruit pulp and seed tissues is greatly influenced by the provenance of origin which was also reported in Mali and Burkina Faso by Parkouda et al. (2007). One possible explanation for the variations detected here is that fruit and seed composition reflects the soil properties of the microsite of each plant (Izhaki, Tsahar, Paluy, & Friedman, 2002). For example, a microsite with a relatively high potassium content in the soil may render production of fruits whose pulp and seeds contain high potassium content. However, further study would be important to verify this theory. In relation to tissue mineral element supply, we may expect contrasting outcomes given that the different provenances inhabit climatically and geologically different habitats that must produce variation in soil nutritional conditions (Fitter & Stickland, 1991). This would support the variation at country level. Also, the availability of nutrients in soil is highly heterogeneous, indicating a strong spatial and temporal variation that is often linked to seasonal and climatic variations, as reported for a Mediterranean shrubland (Monokrousos, Papatheodorou, Diamantopoulos, & Stamou, 2004).

The low variability recorded for most of the macroelements indicates that variation in tissue elemental concentrations was more constrained for nutrients with highest requirements, usually the most abundant and considered the most limiting in nature (Han, Fang, Reich, Ian Woodward, & Wang, 2011). On the contrary, this would suggest that microelements which are the least concentrated in the seed and pulp tissues and less required for plant growth would exhibit high variability within and between provenances. The relatively large variability in pulp and seed mineral concentration among the countries show that plant physiological requirements in conjunction with biogeochemical characteristics influence elemental distribution spatially in soils and biogeographically across climate gradients, as well as constraining general levels of variability (Han et al., 2011). The mineral variations could also be explained by changes in temperature or precipitation which are provoked by climate change ultimately altering nutrient cycles and ecosystems (Matías, Castro, & Zamora, 2011; Sardans & Peñuelas, 2007).

The present results have shown that the order of accumulation of both macro‐ and microelements in pulp and seed tissue is different. For instance, seeds contain higher phosphorus and magnesium than the pulp. This clearly shows that the concentration of mineral elements in these tissues is dependent on a plethora of processes like mobilization, translocation, and redistribution within plant tissues (Singh, Sareen, Sengar, & Kumar, 2013). In east and southern African countries, the least concentrated element for pulp and seeds was manganese, while in West Africa (Mali) it was iron. Furthermore, pulp and seed iron content in Kenyan provenances was three‐ to fivefold the content recorded for Malian samples. This would suggest that the interaction of plant genotype and the environment is responsible for perturbations in the ionome of plant tissue (Baxter et al., 2012). Studies of baobab ionome would provide insights into the plant–environment interaction that influence elemental accumulation since elemental profiles reflect plant adaptation to the environment (Baxter & Dilkes, 2012).

The variation reported here in nutritive traits in baobab wild populations may reflect genetic differentiation (Linhart & Grant, 1996), which is due to a combination of random and selective processes. Such processes lead to ecotypic differentiation as a result of adaptation to local climate (Davis, Shaw, & Etterson, 2005) or environmentally induced phenotypic plasticity (Nicotra et al., 2010). Although it is often difficult to separate trait variation from environmental variation, much of the variation among plant populations is strongly correlated with environmental effects, that is, phenotypic plasticity (Ackerly et al., 2000). Thus, the interprovenance variation in pulp and seed nutritive traits can be inferred to a combination of environmentally induced and genetically fixed differences among baobab populations and may reflect local adaptation or genetic drift leading to differentiation in population traits across climatic gradients. The adaptation would ensure species survival under future climate change.

## 4 CONCLUSION

Baobab fruit contains nutritionally significant levels of essential nutrients including fiber, protein, and minerals. The pulp which can be considered as naturally dry and purely organic food is a rich dietary source of fiber, potassium, calcium, magnesium, iron, and zinc. The levels of these nutrients in the pulp are much higher than those found in commonly consumed fruits such as guava, mango, berry, and bananas. Consumption of the fruit pulp could serve as a potential remedy to reduce micronutrient deficiencies, more so iron and zinc deficiencies, which are prevalent in Africa. Baobab fruits can contribute substantially to local diets and improve the health of local communities. Since the fruits are sold at a low cost, their consumption should be promoted across all regions of Africa.

Overall, there is significant variability in the nutrient content of baobab fruit pulp and seeds from the different provenances across the selected countries. The variation should be exploited during the selection of populations to concentrate on during germplasm collection for use in conservation, breeding programs, and domestication of this “tree of life” ultimately contributing to the expanded use of baobabs beyond SSA. Furthermore, studies should focus on restoring this species and introducing it to novel climates. Since the ICRAF seed bank holds high‐quality baobab seeds, field and laboratory investigations may be carried out to test the ability of the seeds to survive, germinate, and thrive under challenging conditions. Such studies will describe the effects of tree genetics on the nutrient variation because seedlings will be raised in the same environment. As baobab species occur in association with other tree species in agroforestry systems, there will be need to consider the effects arising from species–species interaction which may affect the nutrient levels. Finally, studies need to be conducted to ascertain the effect of soil characteristics on the mineral variability across the different climatic gradients.

## CONFLICT OF INTEREST

The authors declare no conflict of interest.
